# High-Expansion Natural Composite Films for Controlled Delivery of Hydroxycitric Acid in Obesity Therapy

**DOI:** 10.3390/polym17121697

**Published:** 2025-06-18

**Authors:** Kantiya Fungfoung, Ousanee Issarachot, Rachanida Praparatana, Ruedeekorn Wiwattanapatapee

**Affiliations:** 1 Department of Pharmaceutical Technology, Faculty of Pharmaceutical Sciences, Prince of Songkla University, Hat Yai, Songkhla 90112, Thailand; kantiya.1537@gmail.com; 2 Department of Pharmacy Technician, Faculty of Public Health and Allied Health Sciences, Sirindhorn College of Public Health Trang, Praboromrajchanok Institute, 89 M.2 Kantang District, Trang 92110, Thailand; ousanee@scphtrang.ac.th; 3 Faculty of Medical Technology, Prince of Songkla University, Hat Yai, Songkhla 90112, Thailand; rachanida.p@psu.ac.th; 4 Phytomedicine and Pharmaceutical Biotechnology Excellence Center, Faculty of Pharmaceutical Sciences, Prince of Songkla University, Hat Yai, Songkhla 90112, Thailand

**Keywords:** expandable films, chitosan, starch, glucomannan, hydroxycitric acid, gastroretentive drug delivery system, garcinia extract, obesity

## Abstract

Expandable films represent a promising gastroretentive drug delivery system, offering prolonged gastric retention and sustained drug release features particularly advantageous for obesity treatment. This study developed high-expansion films using konjac and various low glycemic index starches, including purple potato, brown rice, resistant, and red jasmine rice starches, in combination with chitosan and hydroxypropyl methylcellulose (HPMC) E15. Garcinia extract was incorporated into the films using the solvent casting technique. Among 27 formulations, all demonstrated rapid unfolding (within 15 min) and significant expansion (2-4 folds). Hydroxycitric acid (HCA), the active component, was encapsulated at efficiencies exceeding 80% *w*/*w*. The konjac-based films exhibited favorable mechanical properties, expansion capacity, and drug content uniformity. Notably, the CK3-H1 formulation (2% *w*/*v* chitosan, 3% *w*/*v* konjac, 1% *w*/*v* HPMC E15) provided sustained HCA release over 8 h via diffusion. Cytotoxicity tests showed no toxic effects on RAW 264.7 macrophages at concentrations up to 400 μg/mL. Furthermore, CK3-H1 achieved notable nitric oxide inhibition (35.80 ± 1.21%) and the highest reduction in lipid accumulation (31.09 ± 3.15%) in 3T3-L1 adipocytes, outperforming pure HCA and garcinia extract. These results suggest that expandable konjac-based films are a viable and effective delivery system for herbal anti-obesity agents.

## 1. Introduction

A gastroretentive drug delivery system (GRDDS) is a novel dosage form designed to remain in the stomach for extended periods, enabling prolonged drug release and enhanced drug absorption and bioavailability [1,2]. One strategy within GRDDS involves the use of expandable films, which are designed to increase in size or volume in response to external or internal stimuli, thereby extending gastric retention time. Recently, expandable films have been successfully employed to deliver active molecules by controlling the release rate and targeting specific sites within the stomach [3]. Nowadays, natural polymers are being researched as alternatives to synthetic ones for the preparation of expandable films due to their biocompatibility, biodegradability, and environmentally friendly properties.

Chitosan is a naturally derived biopolymer that has found widespread application in pharmaceutical formulations. In acidic environments, it forms gel-like structures that prolong gastric retention and facilitate sustained drug release [4]. Consequently, numerous studies have identified chitosan as an effective expandable polymer. Recently, chitosan has been successfully combined with starch as a carrier material for delivering herbal bioactive compounds in the stomach [5,6]. This study reviewed various highly swellable polysaccharides that can be combined with chitosan, including glucomannan (konjac), purple potato starch, brown rice starch, resistant starch, red jasmine rice starch, and glutinous starch, to assess their potential in improving the properties of expandable film formulations. Konjac and most starches excluding glutinous starch have several beneficial properties. These include a low glycemic index (GI), low calorie content, being hypoallergenic, gluten-free, and high in dietary fiber [7]. These features contribute to various health benefits, such as anti-inflammatory [8], antioxidant [9], antimicrobial effects, and antidiabetic effects [10]. As a result, they are well-suited for weight management and can be effectively used to enhance the delivery of herbal medicines in GRDDS.

Glucomannan is a natural polysaccharide derived from the root of the *Amorphophallus konjac* plant. It has attracted considerable attention in oral drug delivery systems due to its ability to absorb large amounts of water, leading to significant swelling and gel formation. However, glucomannan differs markedly from typical starches (such as potato, glutinous, or corn starch) in both water absorption capacity and gelation properties. Specifically, glucomannan can absorb up to 50–100 times its weight in water, forming a very thick, viscous gel. In contrast, starches like corn and potato typically absorb only 2–4 times their weight in water under normal conditions, with viscosity increasing as temperature rises. In terms of gelation mechanisms, glucomannan forms gels via alkali-induced deacetylation, creating networks through hydrogen bonding in a non-thermal process. Starches, on the other hand, undergo heat-dependent gelatinization, typically occurring between 60–80 °C in water. Upon cooling, starches, especially those high in amylose, may form gels through retrogradation [11,12]. In addition to its pharmaceutical applications, glucomannan is widely regarded as a safe dietary supplement, especially for weight control [13,14]. Moreover, plant polysaccharides from various starches were also evaluated. Due to the varying degrees of amylose and the highly branched structure of amylopectin, different starches exhibit different properties such as water absorption, thermal behavior, and gelation [15]. Glutinous rice starch, also known as waxy or sticky starch, contains approximately 80% branched amylopectin. This high amylopectin content contributes to increased water absorption and significant swelling [16]. Brown rice starch is derived from the grain of *Oryza sativa*. This type of starch contains a high amount of amylopectin, ranging from 76.55% to 82.48% [17], which allows it to gelatinize, improve water retention, and enhance film structure [18]. In terms of health benefits, brown rice starch has been studied for its potential to inhibit pancreatic lipase activity [19], support diabetes management, and reduce inflammation [20]. Resistant starch (RS) is a type of dietary fiber that resists digestion in the small intestine and ferments in the colon [21]. It is commonly found in whole grains, potatoes, legumes, and unripe bananas [22]. RS possesses various physicochemical properties that make it suitable for use as a film-forming system. These include ease of retrogradation, strong gelling strength, high swelling capacity and viscosity, and excellent film-forming ability [23]. Interestingly, sweet purple potato starch derived from the *Ipomoea batatas* has been reported to have potential health benefits, including anti-inflammatory effects [8] and antioxidant properties [9]. Moreover, due to its low glycemic index, sweet potato starch can improve insulin sensitivity. Sweet purple potato starch contains about 30% to over 40% of amylose and has unique properties, including biocompatibility and potential for modification, making it a valuable alternative to more commonly used starches like corn starch [24]. Red jasmine rice starch is a variety of Thai rice known for its aromatic and distinctive red bran layer, which contains bioactive compounds such as anthocyanins. Red jasmine rice starch exhibits unique physicochemical properties such as higher water absorption [25] and higher amylose content. These characteristics contribute to its unique pasting and thermal properties, making it suitable for various applications in food and industry [26].

Hydroxycitric acid (HCA) is the main active ingredient in *Garcinia atroviridis* extract. It has been used safely as a dietary supplement for weight management [27]. HCA acts as a competitive inhibitor of ATP citrate lyase during carbohydrate metabolism, thereby preventing fatty acid synthesis. Additionally, it enhances hepatic glycogen synthesis, which contributes to prolonged signaling from the liver to the brain, resulting in the promotion of satiety and helping to suppress appetite for a longer period [28]. However, HCA has some limitations in term of short half-life [29] and undergoes significant first-pass metabolism, impacting its bioavailability and efficacy. Additionally, the commercial products in the market are mostly manufactured in immediate-release dosage forms such as tablets, capsules, gummies and powder, that can lead to concern regarding poor consumer compliance and decrease the efficacy of the treatment [2]. Previous studies have explored encapsulation or matrix-based strategies using lipid-based systems [30] and sustained-release tablets [31] to enhance the bioavailability of hydroxycitric acid (HCA). These delivery systems are capable of protecting HCA from degradation and improving its absorption. However, they typically exhibit a short residence time in the upper gastrointestinal tract, which is the primary site for HCA absorption. To address this limitation, GRDDSs have been proposed, as they can prolong the gastric residence time and enable sustained drug release in the stomach following oral administration.

This research aimed to develop high-expansion natural composite films loaded with garcinia extract that combine chitosan with various polysaccharides, with or without HPMC E15, to achieve sustained release and serve as an optimized carrier for delivering HCA in the stomach. The physicochemical properties of the formulations were thoroughly evaluated. The safety of the film was assessed through cell viability and cell differentiation assays. Finally, the anti-obesity efficacy of the formulation was investigated by examining lipid accumulation and adipogenesis in 3T3-L1 adipocytes.

## 2. Materials and Methods

### 2.1. Materials

Hydroxycitric acid; HCA (purity > 98%) was purchased from MedChemExpress (Bang Trading 1992 Co., Ltd., Bangkok, Thailand). Garcinia extract in powder form, 50% HCA and Konjac powder with 80% glucomannan were provided by AP Operations Co., Ltd. (Chonburi, Thailand). Chitosan flakes were obtained from shrimp shell, with degree of deacetylation (%DD) more than 90% provided by BIO21 Co., Ltd. (Chonburi, Thailand). Methocel E15 premium LV Hydroxypropyl methylcellulose (HPMC E15) was provided by Colorcon Asia Pacific Pte Ltd. (Singapore). Refined glycerine USP was sourced from PC Drug Center Co., Ltd. (Bangkok, Thailand). Glutinous rice starch was from Cho Heng Rice Vermicelli Factory Co., Ltd. (Nakhon Pathom, Thailand). Purple sweet potato powder was obtained from Baan Plai Na Herb Co., Ltd. (Nakhon Pathom, Thailand). Resistant starch (obtained from Cassava) manufactured by BS Starch Chemical Co., Ltd. (Bangkok, Thailand). We purchased organic brown rice starch from Save Life Products Co., Ltd. (Rai Ruen Rom Organic Farm, Chiang Rai, Thailand). Organic red jasmine rice starch was from Fit bakery school (Bangkok, Thailand). Hard gelatin capsules size00 were purchased from Capsugel (Bangkok, Thailand). All other reagents were of analytical grade.

3T3-L1 preadipocyte cells; CL-173^TM^ and RAW264.7 macrophage cells; TIB-71^TM^ were sourced from the American Type Culture Collection (ATCC; Manassas, VA, USA). The following reagents were purchased from Gibco^®^ (Invitrogen, CA, USA): trypan blue solution, penicillin-streptomycin, phosphate buffer saline (PBS), trypsin EDTA 0.25%, DMEM (high glucose Dulbecco’s Modified Eagle Medium), RPMI-1640 (Roswell Park Memorial Institute 1640 medium, and 3-(4,5-dimethyl-2-thiazolyl)-2,5-diphenyl-2H-tetrazolium bromide (MTT reagent). Hyclone^TM^ (Cytiva, Bangkok, Thailand) provided the Fetal bovine serum (FBS). Dimethylsulfoxide (DMSO) was acquired from Amresco^®^ (Solon, OH, USA), while isopropanol was manufactured by Fisher Scientific International, Inc. (Hampton, NY, USA). Formalin was kindly donated by Songklanagarind Hospital (Songkhla, Thailand). Dexamethasone (DEX), 3-isobutyl-1-methylxanthine (IBMX), insulin, Oil Red O dye, indomethacin, and lipopolysaccharide (LPS, from *Escherichia coli*) were purchased from Sigma-Aldrich (Missouri, TX, USA). The whole chemical reagents were of analytical grades.

### 2.2. Preparation of Garcinia Extract-Loaded Expandable Composite Films

Biopolymer-based gastroretentive expandable films were prepared using the solvent casting method. The excipients used in each formulation are listed in Table 1. Various formulations were designed with different concentrations of chitosan (3–4% *w*/*v*), konjac (3–3.5% *w*/*v*), starches (3–3.5% *w*/*v*), HPMC E15 (1–2% *w*/*v*), and glycerin (4% *v*/*v*). Briefly, chitosan was dissolved in 30 mL of acetic acid (1.5% *v*/*v*) and stirred until homogeneous. Then, starches or polysaccharides were added into the chitosan solution under stirring and heated at 80 °C for 1 h to obtain gelatinized starch and form viscous mucilage. Following this, the garcinia extract was added to a viscous mixture and mixed thoroughly to ensure uniform dispersion. Expandable films were cast by pouring them into a glass petri dish plate (area 63.585 cm^2^) and drying them in the hot air oven at 45 °C for 48 h. The obtained films were removed carefully, cut into rectangles size 4 × 2 cm (area 8 cm^2^), then put into hard gelatin capsules (size 00) using a zigzag folding method, kept in a desiccator, and protected from light until use.

### 2.3. Physicochemical Characterization of Garcinia Extract-Loaded Expandable Composite Films

#### 2.3.1. Appearance, Weight Variation, and Thickness of Expandable Films

The appearance of the dried expandable films was recorded in terms of color and surface characterization, and the film samples were placed on the PVC cutting mat and pictures were taken. Weight variation and thickness of films were assessed by cutting each sample to size 4 × 2 cm. The sample’s weight was measured using an analytical balance (PRACTUM224-1S, Sartorius Lab Instruments GmbH & Co., Ltd., Goettingen, Germany), while the thicknesses were measured by using a digital vernier caliper (V6-158 series, Kovet Co., Ltd., Bangkok, Thailand) with an accuracy of ±0.001 mm. The thickness of films was measured before and after an in vitro dissolution test. Each sample was examined in triplicate, and the data were reported as mean ± SD.

#### 2.3.2. Measurement of Mechanical Strength of Expandable Films

The tensile strength of expandable films was measured using a Texture analyzer (TA.XT plus, Stable Micro Systems; Charpa Techcenter Co., Ltd., Bangkok, Thailand) equipped with a 50 kg load cell following the method described by [5]. The film strip, measuring 6 × 1 cm and free of physical imperfections, was fixed between two clamps positioned at a distance of 20 mm. Cardboard was attached to the surface of the clamp to prevent the film from being cut by the clamp grooves. During measurement, the top clamp pulled the strips at a rate of 1 mm/sec until the film broke. The braking force of the films was measured. The tensile strength of films was calculated using Equation (1) as follows. Each formulation was conducted in six replicates, and the tensile strength was reported as the mean ± SD in g/cm^2^.

Tensile strength [g/cm^2^] = force at break (g)/the initial cross-sectional area of the sample (cm^2^) (1)

#### 2.3.3. Swelling Behavior of Expandable Films

The expandable films were cut in size 4 × 2 cm and weighed as initial weight (W_0_). Then, the films were immersed in 0.1 N hydrochloric acid (pH 1.2) and using dissolution apparatus (basket type speed 50 rpm) maintained at a temperature of 37 ± 0.5 °C. Then, the film was removed and weighed again (as W_1_) at different time points: 5, 15, 30, 60, 120, 240, 480 min. The fluid absorption of films was expressed as the percentage of weight gain, calculated using Equation (2):

% Swelling ratio = [(W_1_−W_0_)/W_0_] × 100 (2)

where W_0_ is the initial weight of expandable films, and W_1_ is the weight of expandable films after immersion in 0.1 N hydrochloric acid (pH 1.2) for 480 min. The test was repeated in triplicate and reported as mean ± SD.

#### 2.3.4. Unfolding Behavior of Expandable Films

Dried films of all expandable film formulations were cut with dimension 4 × 2 cm and folded in the zigzag pattern before inserting them into the hard gelatin capsule (size 00). The unfolding behavior was evaluated using an in vitro dissolution basket apparatus in 500 mL of 0.1 N hydrochloric acid (pH 1.2) at 50 rpm. The unfolding of films was observed at intervals of 5, 15, 30, 60, 120, 240, and 480 min. The films in the basket were removed from the vessel at each time interval, and a picture was taken. The unfolding of the films was recorded for 8 h.

#### 2.3.5. Expandable Capacity of Films in Simulated Gastric Fluid

The dimension of the films was recorded to determine the expandable capacity of the films. The films were cut to a size of 4 × 2 cm before testing by the in vitro dissolution test, as mentioned in Section 2.3.3. After a complete time, the dimension of the films was measured again and reported as an expandable area and expansion capacity.

#### 2.3.6. Morphology of Expandable Films

The morphology of the films loaded with garcinia extract was studied by Scanning Electron Microscopy (SEM) (Quanta 400, Hitachi, Tokyo, Japan). The cross-section of films was obtained by bursting into liquid nitrogen and coated with gold prior to investigating an operating accelerating voltage of 15 kV.

#### 2.3.7. X-Ray Diffraction (XRD) Analysis

The X-ray diffraction patterns were analyzed using an X-ray diffractometer (D-5005 diffractometer, Siemens AG, Munich, Germany) at room temperature. The condition of X-ray diffraction studies was under a voltage of 40 kV and a current of 30 mA at a scan speed of 1 s/step over an angles 2θ range 5 to 100° using a step size of 0.05°.

#### 2.3.8. HCA Content Analysis

The expandable films in size 4 × 2 cm were cut and immersed in a 50 mL of 0.1 N hydrochloric acid (pH 1.2) in a volumetric flask followed by sonication (Bandelin sonorex digitec: model DT1028, Becthai Bangkok equipment & chemical Co., Ltd., Bangkok, Thailand) for 30 min. Then, the solution was filtered through a 0.45 µm filter (VertiPure^TM^ PVDF [HL], Vertical Chromatography Co., Ltd., Bangkok, Thailand). The filtrate was taken place to determine the amount of HCA content using UV-visible spectrophotometry at wavelength 210 nm (model; UV-1800, Shimadzu Corporation, Kyoto, Japan). The measurement was done in triplicate for each formulation and data were reported as mean ± SD. The percentage of drug content in the formulations was calculated using Equation (3) as follows:

The percentage of drug content (%) = (A/B) × 100(3)

when A is the actual amount of HCA in the formulation, and B is the initial loaded amount of HCA.

#### 2.3.9. In Vitro Release Studies of HCA from Expandable Films

The release of HCA was performed using the USP dissolution apparatus I (basket type with speed 50 rpm in 500 mL of 0.1 N hydrochloric acid at temperature 37 ± 0.5 °C) (model Varian VK7000, Agilent, Santa Clara, CA, USA). The 5 mL samples were sampled at different time intervals (5, 15, 30, 60, 120, 240, and 480 min) and replaced with fresh dissolution medium in equal volume. The samples were analyzed using the UV-visible method at 210 nm. The drug-release profiles were constructed between time and percent cumulative release of HCA. Each formulation was tested in three replicates.

#### 2.3.10. Release Kinetics

The release of HCA was analyzed against various mathematical models via the DDSolver program [32]. A coefficient of determination (R^2^) value approaching 1 was deemed to demonstrate the best fit of release data to a specific kinetic model.

### 2.4. In Vitro Cell Culture Experiments

#### 2.4.1. In Vitro Cytotoxicity Studies

The cell viability of the expandable films was tested by MTT (3-(4, 5-dimethylthiazol-2-yl)-2-5-diphenyltetrazolium bromide) assay using mouse 3T3-L1 preadipocyte cells and RAW264.7 macrophage cells. The 3T3-L1 and RAW264.7 cells were cultured in DMEM, supplemented with 10% FBS and 1% penicillin-streptomycin at 37 °C under a humidified 5% CO_2_ atmosphere. After, the 3T3-L1 and RAW264.7 cells reached 70% and 90% confluence, respectively. Then, the cells were seeded in a 96-well plate at a density of 5000 and 50,000 cells/well, respectively, and incubated overnight following treatment with serial dilution of standard substance, garcinia extract, blank formulation, and selected expandable film formulation. After 24 h, the cultured medium was removed before adding 0.5 mg/mL MTT solution at 37 °C for 3 h. After that, dimethyl sulfoxide (DMSO) was added to dissolve the formazan product; an absorbance of 570 nm was measured using the microplate reader (Biotek model Power Wave X, Santa Clara, CA, USA). The percentage of cell viability was calculated according to the equation below (4):

Cell viability [%] = [optical density (OD) of sample/optical density (OD) of control] × 100(4)

#### 2.4.2. Anti-Inflammatory Activity Assay

The inhibition of HCA on nitric oxide (NO) production was investigated through a modified method [33]. Cells were seeded (10,000 cells per well) on a 96-well plate and incubated at 37 °C in a 5% CO_2_ atmosphere for 1 h. After this, a fresh medium containing 100 ng/mL of LPS, along with the samples at several concentrations, was replaced, followed by a 24 h incubation. NO production levels were quantified by measuring the nitrile level in the RPMI-1640 medium supernatant using Griess reagent. Optical density was measured at 570 nm (*n* = 5). The percentage inhibition of NO was calculated according to the equation below (5):

The percentage of inhibition of NO production = [(A − B)/(A − C)] × 100(5)

where (A-C) is a NO^−2^ concentration (µM): [A: LPS (+), sample (−)], [B:LPS (+), sample (+)], and [C: LPS (−), sample (−)].

#### 2.4.3. Anti-Obesity Activity in 3T3-L1 Cell Line

Before testing, cell differentiation of 3T3-L1 fibroblasts was evaluated. Differentiation activation medium (D/A) was used to stimulate 3T3-L1 fibroblasts into mature adipocytes, as described in previous studies [34]. Essentially, the differentiation process began by incubating the cells, which were two days post-confluence, in a D/A medium containing 0.5 mM IBMX, 1 µM DEX, and 10 µg/mL insulin (designated as day 0). For day 2, the D/A medium was replaced with the maintenance medium (D/M), which consisted of 10 µg/mL insulin in DMEM high glucose medium, and this medium was changed every 2 days until full adipocyte differentiation was achieved (day 12).

The effects of the samples on the accumulation of lipids in 3T3-L1 cells were evaluated through Oil Red O staining. In summary, 3T3-L1 preadipocytes were seeded in a 48-well plate (20,000 cells per well) and induced to differentiate for 12 days. After this time, the cells were washed with PBS and fixed in a formaldehyde solution (10%) for 60 min and then rinsed twice with 60% isopropanol. Subsequently, Oil Red O solution was added to and incubated for 15 min at room temperature. The lipid staining was visualized under an inverted microscope. The amount of lipid substance was measured after dissolving the cells in 100% isopropanol, with absorbance recorded at 520 nm using a microplate reader [35].

### 2.5. Statistical Analysis

The in vitro study data were reported to standard deviation, and statistical analysis was performed by Student’s *t*-test or one-way analysis of variance (ANOVA). The results of the in vitro cell study are shown as the mean ± SD. The significance of differences was evaluated using one-way ANOVA. Statistical probability (*p*) values less than 0.05 were regarded as significantly different.

## 3. Results and Discussion

### 3.1. Preparation of Garcinia Extract-Loaded Expandable Composite Films

The biopolymer composite films were prepared by the solvent casting approach. The suitability of each polymer concentration was tested. A concentration of 2% *w*/*v* chitosan was selected for its ability to provide suitable viscosity. Subsequently, chitosan at 2% *w*/*v* was combined with different concentrations (3 or 3.5% *w*/*v*) of each starch and konjac. The optimal chitosan/polysaccharides combinations were then selected for the addition of HPMC E15 at 1 or 2% *w*/*v*. HPMC E15 was incorporated to sustain the release of the drug from the composite films. Glycerine used as a film plasticizer and garcinia extract as an active ingredient were fixed at 4% *v*/*v* and 500 mg, respectively.

### 3.2. Physical Appearance of Films

**Figure 1:**
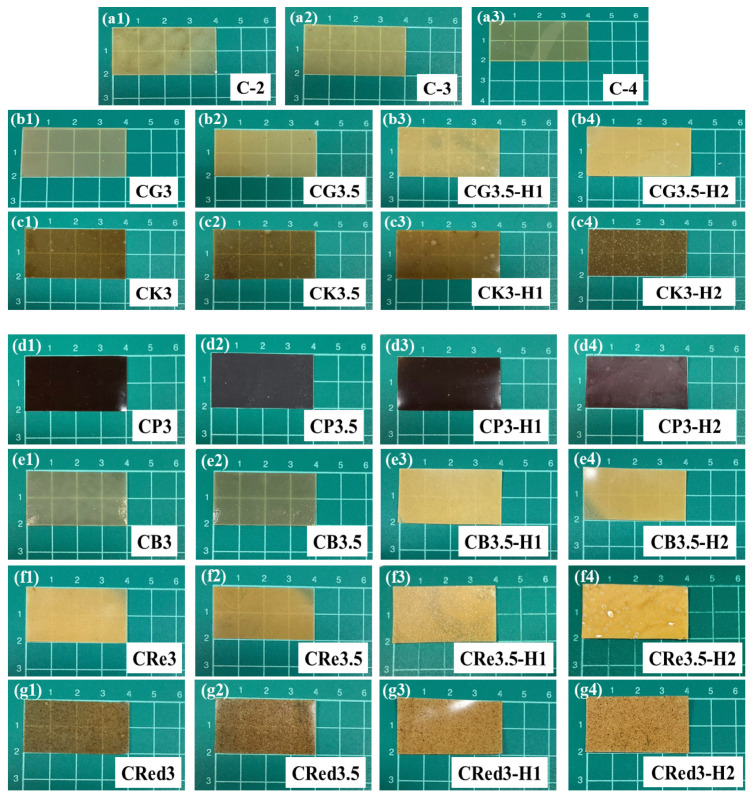
The appearance of various expandable composite film strips, effect of chitosan (**a1**–**a3**), effect of 3% (**b1**–**g1**) and 3.5% (**b2**–**g2**) polysaccharides, and effect of 1% (**b3**–**g3**) and 2% (**b4**–**g4**) HPMC E15.

A total of 27 expandable film formulations were prepared and cut into 4 × 2 cm as shown in Figure 1. Formulations containing only chitosan exhibited a yellow color after oven drying, consistent with the appearance of the chitosan solution (Figure 1(a1–a3)). The glutinous rice starch formulations (Figure 1(b1–b4)) showed a gradual transformation from a transparent film to a turbid one upon the addition of HPMC E15. Similar results were observed for konjac (Figure 1(c1–c4)), brown rice starch (Figure 1(e1–e4)), resistant starch (Figure 1(f1–f4)), and red jasmine rice starch (Figure 1(g1–g4)). In the case of purple potato starch (Figure 1(d1–d4)), the films exhibited a dark purple color across all combinations. These observations can be attributed to the higher solid content in the films and the lower water solubility of HPMC E15 [36,37]. All formulations displayed smooth surfaces without cracking during the drying process and were easily removed from the plates.

### 3.3. Weight and Thickness

The weight of the composite films loaded with garcinia extract ranged from 0.31 ± 0.024 to 0.54 ± 0.006 g as shown in Table 2. The weight of dried films related to the content of the polymer. A similar trend of film weight increment for individual starch represents good reproducibility and homogeneity of the expandable films.

The average film thickness of the dried films ranged from 0.26 ± 0.02 to 0.53 ± 0.02 mm, depending on the formulation’s components, as reflected by the film weight. Chitosan-only formulations showed clearly different expandable film thicknesses, which were related to their concentrations (C-4 > C-3 > C-2). When chitosan was blended with 3 or 3.5% *w*/*v* of each starch and konjac, the slight variation in starch and konjac concentration had a minimal effect on film thickness. The minor increase in their thickness was explained by the higher intermolecular interactions, especially amylose and amylopectin, that formed hydrogen bonds and entanglements, thus creating a denser film structure. This results in less shrinkage and a thicker film after drying and stronger gel formation upon immersion in the acidic environment [38]. Additionally, the higher content of starches enhanced the water retention and gelation of the film [39]. When 1% *w*/*v* of HPMC E15 was added in the formulation, there was no impact to film thickness except at 2% *w*/*v* of HPMC E15.

### 3.4. Mechanical Strength Measurements

The expandable composite films should possess strong mechanical strength to withstand peristalsis in the stomach and are expected to have longer gastric residence time, accompanied by an increase in their dimensions [6,40]. The strength and elasticity of the films were expressed as a tensile strength value, represented in Table 2. The higher chitosan content appeared to significantly decrease tensile strength values of the films (C-2 > C-3 > C-4). This phenomenon can be described by the enhanced hydrogen bonding capability of chitosan, which strengthens polymer interactions. However, when the concentration becomes too high, it may disrupt the uniformity of the polymer network, ultimately resulting in weaker and less cohesive film structures. In some cases, chitosan might reduce the effectiveness of plasticizers such as sorbitol or glycerol in films, making it more rigid and prone to breaking under stress [41,42].

When chitosan was combined with 3 or 3.5% *w*/*v* of glutinous rice starch, purple potato starch, brown rice starch, resistant starch, and red jasmine rice starch, the tensile strengths were increased 2-5 folds in comparison with C-2 film formulation. A particularly dramatic increase in tensile strength was observed for chitosan and konjac (CK3 and CK3.5). The increasing tensile strength values of films can be explained by the high formation of intermolecular hydrogen bonding between NH^3+^ of the chitosan backbone and OH^−^ groups of starches, i.e., in the acetic acid solution, NH_2_ of the chitosan structure protonated to NH^3+^ and molecules of starches were broken down by gelatinization process, resulting in OH^−^ [43]. HPMC E15 is likely to increase the tensile strength of the composite film, especially at 2% *w*/*v* of HPMC E15. This was due to the fact that HPMC E15 can also form a strong, interconnected polymer network through hydrogen bonding [44]. Moreover, the OH^−^ group of HPMC E15 can improve flexibility by reducing brittleness, which enhances the tensile strength and elasticity of the expandable film, and HPMC E15 can act as a plasticizer during film formation. In contrast, the tensile strength of glutinous rice starch films decreased after the addition of HPMC E15. Among the formulations, the konjac film series consistently exhibited the highest tensile strength, suggesting that konjac may help maintain the film’s structural integrity in the gastric environment.

### 3.5. Unfolding Behavior of Films

**Figure 2:**
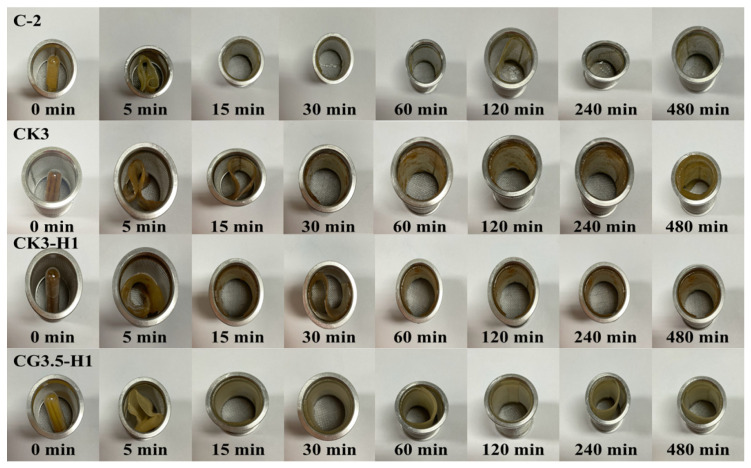

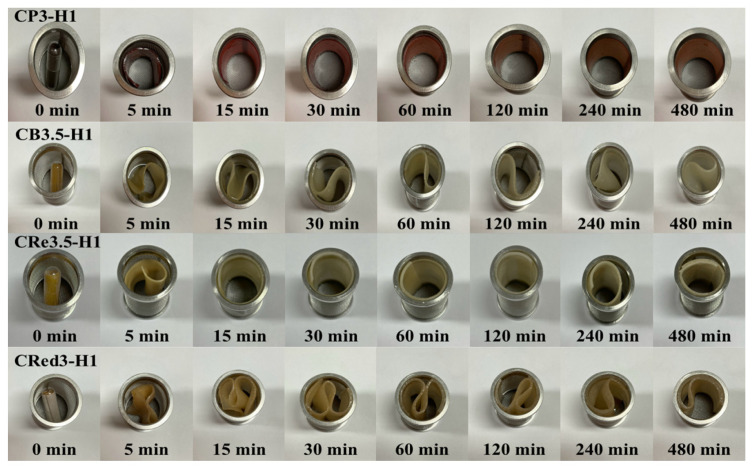
Unfolding behavior of a representative expandable film (*n* = 3).

The unfolding behavior of 27 formulations of expandable composite films was tested in 0.1 N hydrochloric acid (pH 1.2). The capsules, containing films folded in a zigzag pattern, were immersed in the acidic medium. As shown in Figure 2, the capsules fully dissolved within 5 min, allowing the films to completely unfold within 15 min. Additionally, the zigzag films rapidly returned to their original shape upon being immersed in acidic medium. The films maintained their original flat shape for up to 8 h and unfolded without any signs of cracking. Similar unfolding behavior was observed across all formulations. Therefore, one representative image from each series was selected to illustrate the unfolding behavior of konjac and various types of starch [3,5].

### 3.6. Swelling Behavior of Films

**Figure 3:**
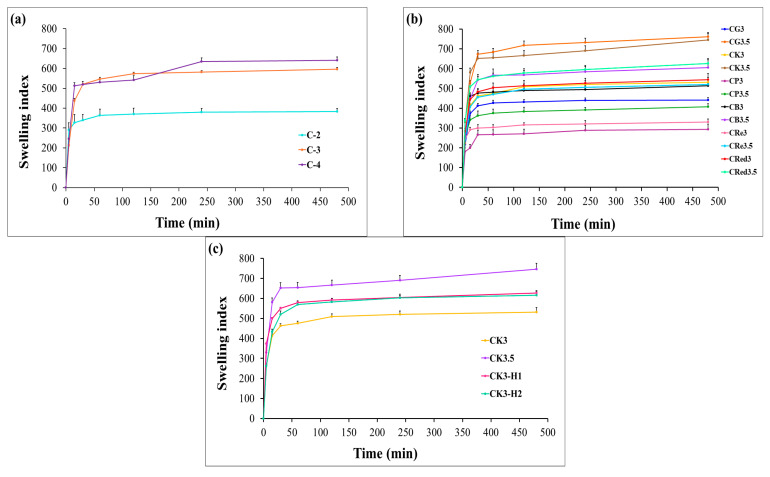
Swelling behavior of expandable composite films containing effect of chitosan concentration (**a**), effect of polysaccharides concentration (**b**), and effect of HPMC E15 concentration in konjac series (**c**). Bars represent mean ± SD (*n* = 3).

The swelling properties represent the capacity of hydrophilic polymers to absorb water. To evaluate this behavior, the films were weighed before and after immersion in 0.1 N hydrochloric acid (pH 1.2) for 8 h. The swelling index was calculated using equation 2. Chitosan demonstrated enhanced swelling capacity with increasing concentration (C-4 > C-3 > C-2) as illustrated in Figure 3a. The swelling behavior of chitosan-based films is influenced by the concentration of chitosan in a dose-dependent manner. The higher swelling index observed with increasing chitosan concentration may be attributed to the hydrophilic nature of chitosan and the availability of more functional groups (such as –OH and –NH_2_) that interact with water molecules. At higher concentrations, the film matrix contains more chitosan chains, which can increase the number of water-binding sites, enhance water absorption and lead to greater swelling. The combination of chitosan with lower amounts of starches, such as glutinous rice starch, purple potato starch, brown rice starch, resistant starch, and red jasmine starch, resulted in a reduced swelling index of the films. However, a higher concentration of starch (3.5% *w*/*v*) can improve the swelling index. These results can be explained by the disruption of the chitosan crystalline structure, which creates a more heterogeneous polymer matrix in expandable films. Additionally, starch represented hydrophilic nature and was able to absorb water primarily due to the presence of amylopectin [45]. As can be seen in Figure 3b, the saturated water absorption for each formula occurred within approximately 30 min and slightly increased up to 8 h. As shown in Table 3, the swelling index increased after the combination with 1% and 2% HPMC E15. However, there was no significant difference in the swelling values between the two concentrations. The higher content of konjac showed a larger swelling index (CK3.5 > CK3). The combination of CK3 with 1% or 2% HPMC E15 had a higher swelling index (Figure 3c). This was due to HPMC E15, which can promote the water-holding capacity and gel-forming ability [46].

### 3.7. Measurement of Expansion Ability

**Figure 4:**
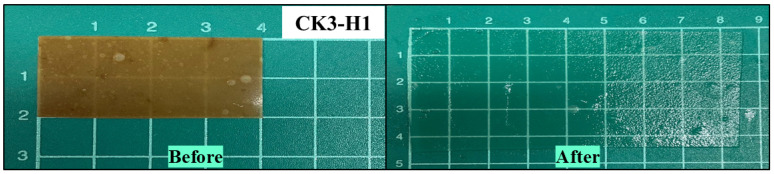
The characteristics of expandable composite films based on konjac (CK3-H1) before and after the dissolution test (*n* = 3).

The expansion capacity of the films was represented in term of expandable area and expansion capacity after the dissolution test for 8 h. The size of the dried films and immersed films were measured using a digital vernier caliper. Chitosan-based films exhibited expansion depending on the chitosan concentration as mentioned in Table 3. When combined with starches such as glutinous rice starch, purple potato starch, brown rice starch, resistant starch, and red jasmine rice starch, the films expanded approximately 2–4 fold. Similar results were obtained after the addition of HPMC E15. Remarkably, the high expansion capacity was observed for konjac with CK3 (4.32 ± 0.13 folds), CK3-H1 (4.32 ± 0.06 folds), and CK3-H2 (4.12 ± 0.13 folds), respectively, while the formulation based on glutinous starch (CG3.5) revealed the highest expansion capacity (4.52 ± 0.27 folds). The highly expandable area of formulations containing glutinous rice starch may be attributed to their high amylopectin content [6,47]. As seen in Figure 4, the characteristics of expandable film formulation based on konjac before and after the dissolution test are presented.

### 3.8. Scanning Electron Microscopy (SEM) Studies

**Figure 5:**
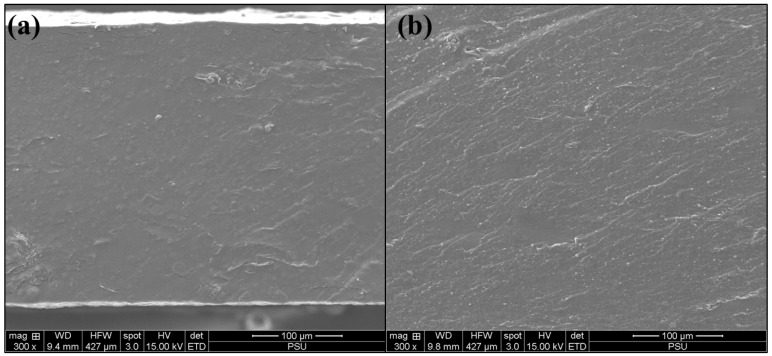
The cross-sectional view of blank CK3-H1 (**a**) and CK3-H1 formulation (**b**) at 300× magnification.

The Blank CK3-H1 film (Figure 5a) and CK3-H1 (Figure 5b) formulations were examined for the surface morphology of the composite films. According to Figure 5, the cross-sectional views revealed a rough, non-porous structure without the presence of surface crystals, indicating a homogeneous dispersion of garcinia extract within the polymer matrix. No significant differences in surface morphology were observed between the blank and CK3-H1 formulations.

### 3.9. X-Ray Diffraction (XRD) Studies

**Figure 6:**
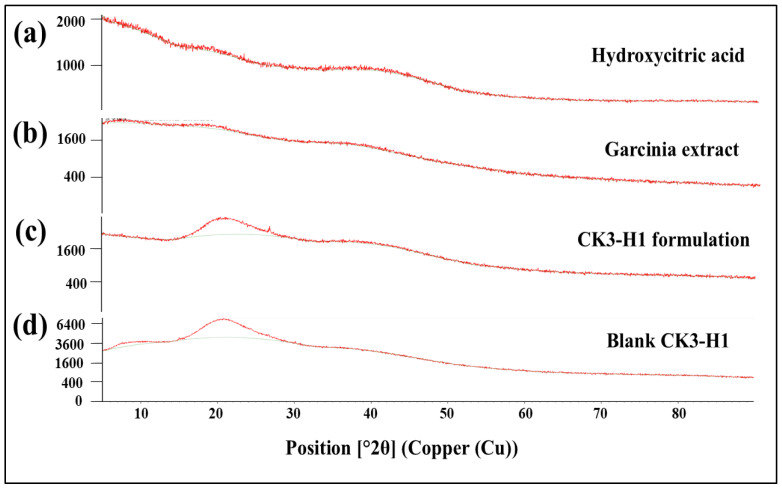
X-ray diffraction patterns of hydroxycitric acid standard (**a**), garcinia extract (**b**), CK3-H1 formulation (**c**), and blank CK3-H1 expandable film (**d**).

XRD equipment was used to detect the crystalline and amorphous form of the drug. The diffractograms of the hydroxycitric acid standard compound, garcinia extract, blank film, and CK3-H1 formulation are illustrated in Figure 6. The HCA standard revealed an amorphous form, as no sharp peaks were observed in the diffractogram (Figure 6a). The same phenomenon was observed for garcinia extract (Figure 6b), CK3-H1 formulation (Figure 6c), and blank CK3-H1 film (Figure 6d). These results indicated that HCA did not change its crystallinity during film preparations.

### 3.10. Drug Content Uniformity

The hydroxycitric acid (HCA) content in expandable composite film formulations was measured by using UV-visible spectrophotometer. The percentage of drug content of each formulation was in the range of 80.51% to 98.27% as shown in Table 2. This result demonstrated that expandable films are capable of entrapping high amounts of HCA.

### 3.11. In Vitro Dissolution Testing

**Figure 7:**
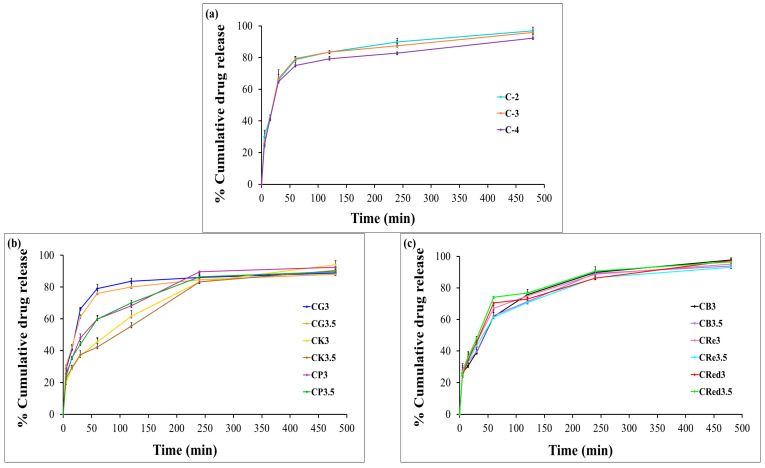

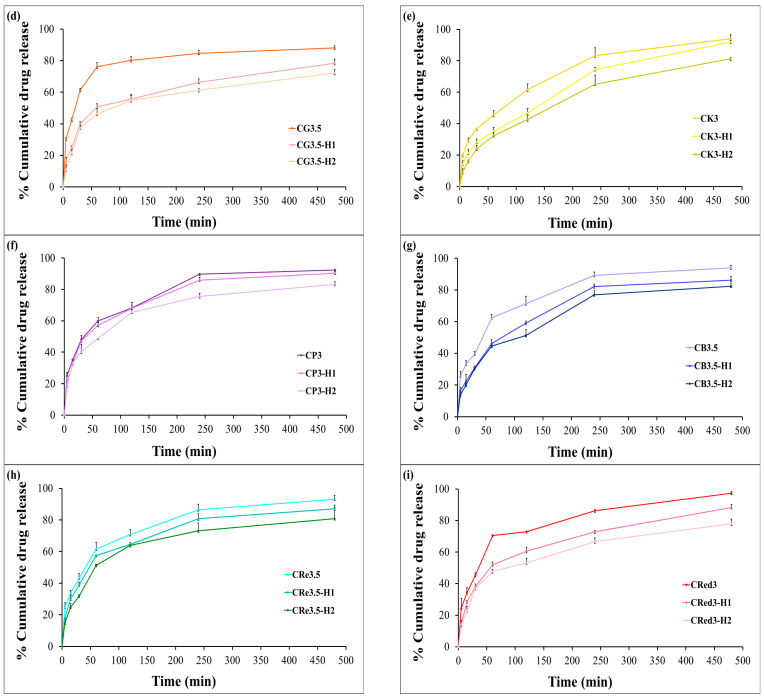
The release profiles of the expandable composite film in 0.1 N hydrochloric acid (pH 1.2): effect of chitosan concentration (**a**), effect of konjac and different types of starch (**b**,**c**), and effect of HPMC E15 in the formulations (**d**–**i**). Data are presented as mean ± SD (*n* = 3).

The cumulative release of HCA from the expandable films was evaluated using dissolution testing. In the presence of chitosan alone, HCA exhibited a burst release of approximately 80% from chitosan-based films within the first hour (Figure 7a). The effect of various polysaccharide composite films on the release profiles of HCA are shown in Figure 7b,c. The results indicated that glutinous rice starch was ineffective in retarding HCA release. In contrast, purple potato starch, brown rice starch, resistant starch, and red jasmine rice starch were able to reduce the burst release to 60–70% within the first hour (illustrated in Figure 7d–i).

Interestingly, the expandable films based on konjac demonstrated a more sustained release of HCA compared to the other starches. Furthermore, varying the concentration of HPMC E15 significantly reduced the initial burst release of HCA compared to formulations without HPMC. Among the tested formulations, CK3-H1 exhibited the most desirable release profile, characterized by a sustained release of over 80% of HCA within 8 h (Figure 7e). This behavior could be explained by the high degree of polymer chain entanglement in HPMC, allowing it to act as a release retardant, thereby limiting the diffusion of the active compound from the dosage form [48]. The drug release from swellable hydrophilic polymers can be controlled by the physical and chemical structure of polymers, solvent penetration into the polymer network, drug diffusion throughout the swollen matrix, and the swelling and erosion of hydrated polymers. CK3-H1 formulation (2% chitosan, 3% konjac, 1% HPMC E15, and 500 mg garcinia extract) was selected to be the optimized expandable film formulation due to it having the suitable physicochemical properties and also enhancing the prolonged release.

### 3.12. Release Kinetics Pattern

The drug release from konjac–chitosan-based formulations were selected to fit with correlation coefficient (R^2^), release exponent (*n*), and shape parameter (β) values using the various kinetic models. The R^2^ values were used to evaluate the best fit model for each formulation and describe the release mechanism. As represented in Table 4, the release of HCA fitted with the Higuchi model, which describes drug release as a diffusion process based on Fick’s law, and R^2^ values were more than 0.89, especially for CK3-H1 (0.9828) and CK3-H2 (0.9905). These results imply that diffusion from matrices was a major mechanism in the release process [49]. The Weibull model demonstrated strong fitting for formulations (R^2^ > 0.96), and the β values were not more than 0.75, suggesting that Fickian diffusion from the polymeric network within film structure by initial burst release of HCA followed by slower release, typical of matrix systems. Additionally, the drug release data followed the Korsmeyer–Peppas model and showed excellent fitting (R^2^ > 0.98), with release exponent “*n*” values < 0.45, indicating a Fickian diffusion mechanism (drug release dominated by diffusion). The formulation exhibited sustained release over 8 h. The formulations (CK3-H1 and CK3-H2) showed more sustained release (higher *n* and β values) and are better candidates for controlled delivery systems than CK3 and CK3.5 formulations.

Among the tested models, the Korsmeyer–Peppas and Higuchi models provided the greatest values of R^2^, suggesting that diffusion plays role in the release mechanism, particularly for the CK3-H1 and CK3-H2 formulations, as evidenced by *n* values less than 0.45. These results also indicate that the incorporation of HPMC enhances controlled drug release, likely by modulating the matrix structure and altering diffusion pathways [50].

### 3.13. Cytotoxicity Assay

**Figure 8:**
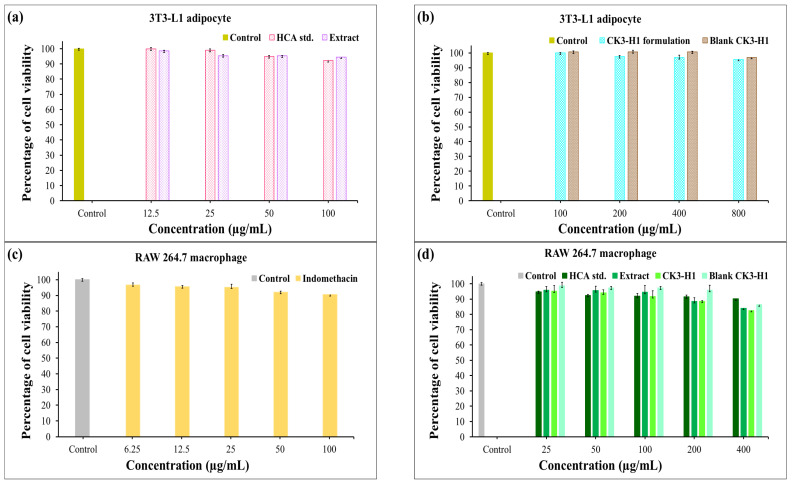
Cell viability of 3T3-L1 (**a**,**b**) and RAW 264.7 macrophage cells (**c**,**d**) following exposure for 24 h and treated with hydroxycitric acid standard, garcinia extract, blank CK3-H1 expandable film, and CK3-H1 expandable film loaded with garcinia extract. Data reported as mean ± SD (*n* = 5).

The results represented the effects of different treatments and various concentrations on the viability of 3T3-L1 adipocyte and RAW 264.7 macrophage cells, likely assessed post 24 h treatment using MTT assay, which is a standard method for evaluating cytotoxicity in response to various stimuli [51]. The control group was used as a baseline to compare cell viability across the treatment groups. For 3T3-L1 cells, no significant differences were observed between the treatment groups and the control (*p* > 0.05). Treatments include the HCA standard, garcinia extract, CK3-H1 formulation, and blank CK3-H1. All demonstrated high percentages of cell viability, indicating very low toxicity toward 3T3-L1 adipocytes. Notably, the cells remained viable even at high concentrations (800 µg/mL), as shown in Figure 8a,b. Moreover, the MTT reduction test performed on RAW 264.7 cells displayed that cell viability remained above 80% following exposure to increasing concentrations of the test samples (Figure 8c,d). These findings indicate that the compounds and other ingredients in the formulations are non-toxic to both 3T3-L1 adipocytes and RAW 264.7 macrophage cells.

### 3.14. Anti-Inflammatory Assay

The anti-inflammatory effect was evaluated by measuring the amount of nitric oxide (NO), a common inflammatory marker. Lipopolysaccharide (LPS), a component of Gram-negative bacterial cell walls, was used as a positive control due to its ability to elicit a strong inflammatory response in RAW 264.7 cells. It activates TLR4 (Toll-like receptor 4), which then induces NF-κB (nuclear factor kappa B) and MAPK (mitogen-activated protein kinase) pathways. This leads to an increase in NO production via iNOS cytokine secretion and COX-2 expression [52]. The anti-inflammatory activities of the HCA standard, garcinia extract, CK3-H1 formulation, and CK3-H1 blank were evaluated and compared with indomethacin as a reference drug.

The expandable composite film loaded with garcinia extract (CK3-H1 formulation; equivalent to 108 µg of HCA) moderately inhibited NO production in RAW 264.7 cells, with an inhibition rate of 35.80 ± 1.21%, as shown in Table 5. In comparison, indomethacin at a concentration of 50 µg/mL exhibited a significantly higher NO inhibition of 52.90 ± 2.60%. Remarkably, the blank CK3-H1 formulation exhibited only a weak ability to inhibit NO production in RAW 264.7 cells (14.23 ± 0.84%), which can be attributed to the absence of HCA in the film. These findings confirm that the CK3-H1 formulation possesses a notable anti-inflammatory effect, although its efficacy is lower than that of indomethacin. Furthermore, anti-inflammatory activity is closely associated with lipid accumulation in adipocytes, suggesting potential dual-action benefits of the formulation. Reducing inflammation can improve insulin sensitivity and normalize adipocyte function [53]. Adipocytes play a key role in metabolic inflammation, particularly in obesity-related conditions where excessive lipid accumulation promotes immune cells to secrete pro-inflammatory cytokines like TNF-α, IL-6, and MCP-1 (monocyte chemoattractant protein-1). This also makes anti-inflammatory strategies a promising approach for managing obesity and associated metabolic syndromes.

### 3.15. Lipid Accumulation Assay

**Figure 9:**
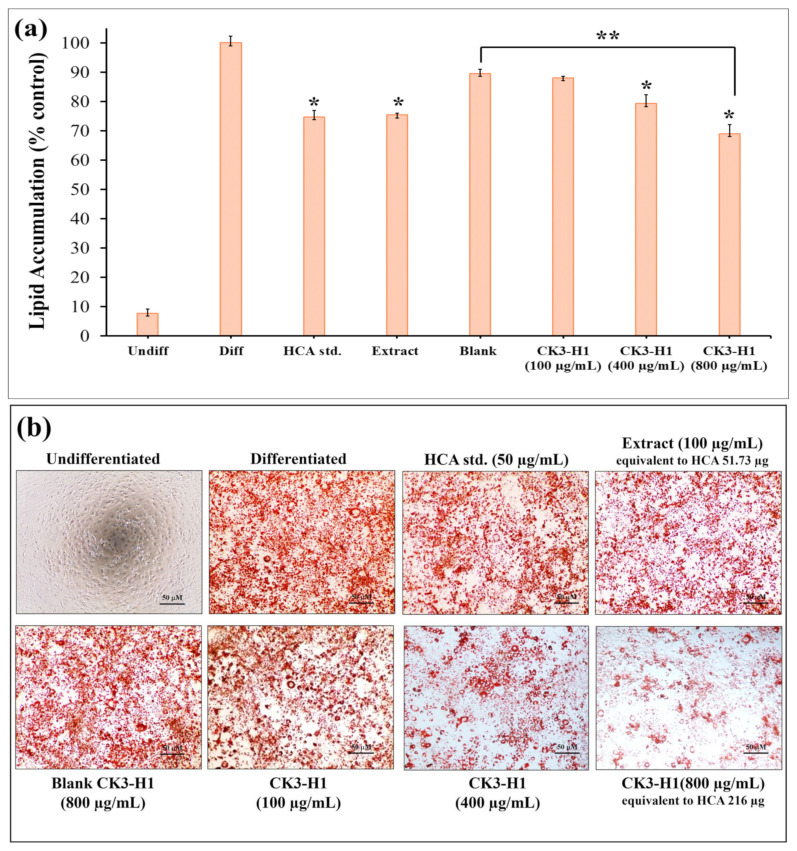
The effect of lipid droplet formation in 3T3-L1 adipocytes treated with various concentrations of HCA standard, garcinia extract, CK3-H1 formulation, and blank CK3-H1, as assessed by Oil Red O staining: (**a**) The percentage of lipid accumulation in 3T3-L1 adipocytes across different sample groups is illustrated, and (**b**) representative cell images were captured. Data are expressed as the mean ± SD. * *p* < 0.05 compared to differentiated group, ** *p* < 0.05 compared with blank CK3-H1 formulation.

Investigating lipid accumulation in 3T3-L1 cells typically involves several key pathways and methods. Once 3T3-L1 cells were fully differentiated into adipocytes, lipid accumulation was observed, and the lipid content was quantified by eluting the dye with isopropanol and measuring absorbance at 520 nm. The undifferentiated group (negative control) showed barely any lipid accumulation (7.65 ± 1.54%), indicating the absence of adipocyte differentiation. On the other hand, the differentiated group (positive control) exhibited nearly complete lipid accumulation (100 ± 2.17%), consistent with full adipocyte maturation as shown in Figure 9a. Treatments with the HCA standard, garcinia extract, and CK3-H1 formulation significantly reduced lipid accumulation in 3T3-L1 adipocytes compared with the differentiated group. The corresponding lipid accumulation percentages were 74.66 ± 2.27%, 75.34 ± 0.59%, and 68.91 ± 3.15%, respectively. Moreover, CK3-H1 (concentration 800 µg/mL) demonstrated significantly reduced lipid content when compared with blank formulation at the same concentration. Additionally, Oil Red O staining technique was used to visualize lipid accumulation by binding dye to lipid droplets in cell and staining them red as shown in Figure 9b. The results demonstrated that CK3-H1 formulation significantly reduced adipocyte differentiation in a concentration-dependent manner. Furthermore, HCA standard and garcinia extract also showed the reduction of lipid content in 3T3-L1 adipocytes compared with the differentiated group. These findings support the conclusion that garcinia extract-loaded expandable films effectively inhibit lipid accumulation and significantly suppress adipogenesis in 3T3-L1 adipocyte cells [54].

## 4. Conclusions

In this study, high-expansion gastroretentive composite films incorporating Garcinia extract were successfully developed using chitosan blended with konjac and high-swelling starches, including brown rice starch, resistant starch, and red jasmine rice starch. These films exhibited favorable properties for gastric retention, such as high expandability, unfolding capability, and effective swelling behavior. Among the tested formulations, the chitosan film containing 3% *w*/*v* konjac and 1% *w*/*v* HPMC E15 (CK3-H1) demonstrated optimal mechanical strength and a sustained release profile, delivering over 80% of hydroxycitric acid (HCA) within 8 h. Notably, CK3-H1 also significantly inhibited adipogenesis in 3T3-L1 cells, indicating its biological efficacy. These findings underscore the therapeutic potential of expandable, konjac-based gastroretentive films loaded with Garcinia extract as a novel and effective strategy for herbal-based obesity management. Future work will focus on comprehensive stability testing under various storage conditions to evaluate shelf life and packaging requirements. Additionally, preclinical and clinical studies will be necessary to validate the formulation’s efficacy and safety profile, supporting its advancement toward practical therapeutic applications.

## Figures and Tables

**Table 1:** The composition of expandable composite film formulations.

Ingredient Concentration (% *w*/*v*) *								
Batch Code	Chitosan	Glutinous Rice Starch	Konjac	Purple Potato Starch	Brown Rice Starch	Resistant Starch	Red Jasmine Rice Starch	HPMC E15
*Effect of Chitosan*								
C-2	2	-	-	-	-	-	-	-
C-3	3	-	-	-	-	-	-	-
C-4	4	-	-	-	-	-	-	-
*Effect of Polysaccharides*								
CG3	2	3	-	-	-	-	-	-
CG3.5	2	3.5	-	-	-	-	-	-
CK3	2	-	3	-	-	-	-	-
CK3.5	2	-	3.5	-	-	-	-	-
CP3	2	-	-	3	-	-	-	-
CP3.5	2	-	-	3.5	-	-	-	-
CB3	2	-	-	-	3	-	-	-
CB3.5	2	-	-	-	3.5	-	-	-
CRe3	2	-	-	-	-	3	-	-
CRe3.5	2	-	-	-	-	3.5	-	-
CRed3	2	-	-	-	-	-	3	-
CRed3.5	2	-	-	-	-	-	3.5	-
*Effect of HPMC E15*								
CG3.5-H1	2	3.5	-	-	-	-	-	1
CG3.5-H2	2	3.5	-	-	-	-	-	2
CK3-H1	2	-	3	-	-	-	-	1
CK3-H2	2	-	3	-	-	-	-	2
CP3-H1	2	-	-	3	-	-	-	1
CP3-H2	2	-	-	3	-	-	-	2
CB3.5-H1	2	-	-	-	3.5	-	-	1
CB3.5-H2	2	-	-	-	3.5	-	-	2
CRe3.5-H1	2	-	-	-	-	3.5	-	1
CRe3.5-H2	2	-	-	-	-	3.5	-	2
CRed3-H1	2	-	-	-	-	-	3	1
CRed3-H2	2	-	-	-	-	-	3	2

* The amount of glycerin and garcinia extract in each formulation was fixed at 4% *v*/*v* and 500 mg, respectively.

**Table 2:** The physicochemical characteristics of expandable composite films.

Batch code		Weight (g)	Thickness (mm)	Tensile Strength (g/cm^2^)	Drug Content (%)
Chitosan	C-2	0.31 ± 0.024	0.26 ± 0.02	34.93 ± 2.067	98.27 ± 0.42
	C-3	0.37 ± 0.013	0.35 ± 0.04	27.71 ± 3.115	93.38 ± 4.56
	C-4	0.42 ± 0.008	0.48 ± 0.01	22.61 ± 1.351	92.85 ± 3.06
Glutinous rice starch	CG3	0.38 ± 0.018	0.30 ± 0.02	54.19 ± 2.313	92.72 ± 2.93
	CG3.5	0.45 ± 0.025	0.39 ± 0.03	90.23 ± 6.338	88.03 ± 1.97
	CG3.5-H1	0.48 ± 0.027	0.46 ± 0.03	59.80 ± 3.046	86.85 ± 1.31
	CG3.5-H2	0.52 ± 0.017	0.48 ± 0.03	74.44 ± 7.738	85.60 ± 3.01
Konjac	CK3	0.41 ± 0.012	0.40 ± 0.02	155.80 ± 7.581	94.46 ± 1.79
	CK3.5	0.43 ± 0.015	0.46 ± 0.04	214.82 ± 6.011	87.87 ± 2.19
	CK3-H1	0.45 ± 0.027	0.45 ± 0.02	382.95 ± 2.137	92.94 ± 1.29
	CK3-H2	0.51 ± 0.008	0.50 ± 0.01	625.81 ± 4.810	81.56 ± 1.64
Purple potato starch	CP3	0.42 ± 0.015	0.39 ± 0.03	57.14 ± 3.242	95.09 ± 3.62
	CP3.5	0.43 ± 0.014	0.40 ± 0.04	71.55 ± 4.455	87.14 ± 1.64
	CP3-H1	0.46 ± 0.021	0.46 ± 0.02	78.03 ± 7.860	92.15 ± 1.20
	CP3-H2	0.51 ± 0.006	0.52 ± 0.03	86.43 ± 1.860	89.78 ± 3.36
Brown rice starch	CB3	0.44 ± 0.018	0.42 ± 0.03	51.87 ± 2.962	86.77 ± 2.56
	CB3.5	0.47 ± 0.001	0.42 ± 0.03	124.68 ± 3.213	90.20 ± 2.81
	CB3.5-H1	0.48 ± 0.013	0.42 ± 0.02	132.10 ± 7.245	90.16 ± 2.86
	CB3.5-H2	0.52 ± 0.020	0.51 ± 0.02	166.13 ± 2.108	87.20 ± 3.29
Resistant starch	CRe3	0.44 ± 0.008	0.40 ± 0.02	88.68 ± 2.515	84.46 ± 1.43
	CRe3.5	0.48 ± 0.014	0.43 ± 0.04	108.12 ± 4.877	89.40 ± 1.52
	CRe3.5-H1	0.51 ± 0.031	0.43 ± 0.03	115.08 ± 1.333	83.23 ± 2.72
	CRe3.5-H2	0.53 ± 0.006	0.53 ± 0.02	162.73 ± 2.220	81.77 ± 2.46
Red jasmine rice starch	CRed3	0.41 ± 0.028	0.43 ± 0.04	53.07 ± 1.052	88.41 ± 3.58
	CRed3.5	0.43 ± 0.022	0.46 ± 0.02	69.34 ± 1.970	84.08 ± 1.96
	CRed3-H1	0.46 ± 0.019	0.46 ± 0.02	74.50 ± 9.998	86.56 ± 1.66
	CRed3-H2	0.51 ± 0.032	0.54 ± 0.02	149.60 ± 4.834	80.51 ± 2.79

**Table 3:** Swelling index and expandable properties of expandable composite films.

Batch Code		Swelling Index	Expandable Area (cm^2^)	Expansion Capacity * (Fold)
Chitosan	C-2	382.85 ± 16.09	24.83 ± 1.32	3.10 ± 0.17
	C-3	596.66 ± 8.15	28.61 ± 1.14	3.58 ± 0.14
	C-4	641.15 ± 16.04	31.21 ± 1.04	3.90 ± 0.13
Glutinous rice starch	CG3	440.77 ± 11.93	23.46 ± 0.34	2.93 ± 0.04
	CG3.5	760.73 ± 22.72	36.14 ± 2.13	4.52 ± 0.27
	CG3.5-H1	777.45 ± 29.04	27.01 ± 0.37	3.41 ± 0.09
	CG3.5-H2	801.94 ± 12.22	29.64 ± 0.39	3.90 ± 0.21
Konjac	CK3	531.00 ± 23.06	34.57 ± 1.02	4.32 ± 0.13
	CK3.5	745.95 ± 30.07	27.00 ± 2.19	3.38 ± 0.27
	CK3-H1	627.70 ± 5.88	34.57 ± 0.47	4.32 ± 0.06
	CK3-H2	616.21 ± 22.90	31.32 ± 1.57	4.12 ± 0.13
Purple potato starch	CP3	293.09 ± 26.90	15.31 ± 0.42	1.91 ± 0.05
	CP3.5	407.36 ± 15.80	16.05 ± 0.32	2.01 ± 0.04
	CP3-H1	410.67 ± 23.79	15.68 ± 0.28	2.05 ± 0.11
	CP3-H2	552.26 ± 27.68	18.60 ± 0.31	2.39 ± 0.11
Brown rice starch	CB3	513.12 ± 29.36	21.01 ± 0.18	2.63 ± 0.02
	CB3.5	604.98 ± 40.18	23.22 ± 0.20	2.90 ± 0.02
	CB3.5-H1	651.56 ± 27.67	27.38 ± 0.98	3.51 ± 0.12
	CB3.5-H2	679.82 ± 16.91	25.43 ± 0.56	3.26 ± 0.08
Resistant starch	CRe3	330.43 ± 15.79	18.62 ± 1.34	2.33 ± 0.17
	CRe3.5	519.50 ± 7.55	19.75 ± 1.01	2.55 ± 0.04
	CRe3.5-H1	615.96 ± 17.57	19.84 ± 0.95	2.48 ± 0.12
	CRe3.5-H2	641.60 ± 15.62	19.32 ± 0.79	2.57 ± 0.21
Red jasmine rice starch	CRed3	544.32 ± 31.28	22.11 ± 0.33	2.86 ± 0.09
	CRed3.5	625.93 ± 25.96	20.69 ± 0.49	2.63 ± 0.03
	CRed3-H1	600.89 ± 21.07	28.62 ± 0.56	3.58 ± 0.07
	CRed3-H2	639.83 ± 12.17	25.80 ± 1.05	3.40 ± 0.25

* Expansion capacity was compared with dried films.

**Table 4:** Correlation coefficient of konjac-chitosan expandable film series.

Batch Code	Kinetic Release Models							
	Zero Order (R^2^)	First Order (R^2^)	Higuchi (R^2^)	Hixson–Crowell (R^2^)	Korsmeyer–Peppas (R^2^)	*n*	Weibull (R^2^)	β
CK3	0.2977	0.8973	0.9119	0.8508	0.9915	0.344	0.9870	0.587
CK3.5	0.2605	0.8437	0.8902	0.7988	0.9800	0.335	0.9679	0.544
CK3-H1	0.6306	0.9301	0.9828	0.9029	0.9911	0.440	0.9782	0.753
CK3-H2	0.6682	0.9309	0.9905	0.8864	0.9945	0.458	0.9941	0.668

**Table 5:** Anti-inflammatory activity of hydroxycitric acid standard, garcinia extract, blank, and optimized formulation (CK3-H1). Data reported as mean ± SD.

Samples	The Percentage of Nitric Oxide Inhibition
Indomethacin conc. 50 µg/mL	52.90 ± 2.60
HCA std. conc. 100 µg/mL	27.67 ± 0.23
Extract conc. 200 µg/mL (equivalent to HCA 103.46 µg)	26.94 ± 1.05
CK3-H1 formulation conc. 400 µg/mL (equivalent to HCA 108 µg)	35.80 ± 1.21
Blank CK3-H1 formulation conc. 400 µg/mL	14.23 ± 0.84

## Data Availability

Data will be made available on request.
